# Integration of resistance exercise into a multimodal approach to prehabilitation for patients with sarcopenia prior to surgery: a narrative review

**DOI:** 10.3389/fresc.2025.1481233

**Published:** 2025-05-13

**Authors:** Harsh Patel, Khang Duy Ricky Le, Annie Jiao Wang, Samuel Boon Ping Tay

**Affiliations:** ^1^ Department of General Surgical Specialties, The Royal Melbourne Hospital, Parkville, VIC, Australia; ^2^ Geelong Clinical School, Deakin University, Geelong, VIC, Australia; ^3^ Department of Surgical Oncology, The Peter MacCallum Cancer Centre, Parkville, VIC, Australia; ^4^ Department of Anaesthesia and Pain Medicine, Box Hill Hospital, Eastern Health, Box Hill, VIC, Australia

**Keywords:** prehabilitation, rehabilitation, sarcopenia, resistance exercise, perioperative medicine, perioperative optimisation

## Abstract

**Introduction:**

Sarcopenia describes the process of progressive, generalised loss of skeletal muscle mass and strength, and has been recognised as a predictor of postoperative complications and mortality. Prehabilitation represents a clinical strategy where patients undergo both physical and psychological strategies in order to improve their functional capacity prior to surgery. Importantly, prehabilitation programs have been considered as an area of perioperative optimisation to address sarcopenia. However, the optimal prehabilitation program regimen remains poorly characterised. Instead of suggesting a novel prehabilitation strategy for sarcopenic patients, this review seeks to characterise the best-practice modalities and methods of resistance training as a component of multimodal prehabilitation to improve patient outcomes following surgery.

**Methods:**

A narrative review was performed following a search of Medline and Embase databases.

**Results:**

There is significant heterogeneity in the literature regarding best-practive resistance exercise regimens for patients with sarcopenia who are awaiting surgery. Overall, the literature highlights that programs with early involvement of clinicians, dietitians, nutritionists, and psychological support programs have been shown to improve patient outcomes compared to programs that did not. Additionally, asides from muscular hypertrophy, resistance exercise programs have been shown to have a multifactorial impact on sarcopenia, synergistically improving the domains of nutrition, mental health, hormonal imbalance, and chronic inflammation. The ideal approach to resistance exercise remains poorly understood, with a paucity of evidence surrounding the best methods for delivering such regimens. Despite this, key considerations revealed by this review include the need for prehabilitation clinicians to consider key aspects of resistance training including training volume, intensity with consideration into periodisation and progressive overload. Collaboration with multidisciplinary networks such as physiotherapists, exercise physiologists and personal trainers should be considered to ensure a safe and injury-free approach to resistance exercise in prehabilitation.

**Conclusion:**

While there remains a lack of standardisation of prehabilitation protocols, the evidence suggests that multimodal prehabilitation should be considered in evidence-based frameworks to improve patient outcomes following surgery. In particular, the ability of resistance exercises to address multiple domains relevant to sarcopenia, thereby enhancing patient outcomes beyond pure hypertrophy and playing a key role in prehabilitation.

## 1 Introduction

Sarcopenia refers primarily process of progressive, generalised loss of skeletal muscle mass, strength and therefore performance (1). It has been reported that the prevalence of sarcopenia in the general population is 10% (2). Importantly, pre-surgery sarcopenia has recently emerged as a clinically important prognostic marker of postoperative outcomes (3). Its presence has been demonstrated to be associated with an increased risk of poorer outcomes. Specifically, pre-surgery sarcopenia has been shown to be a marker of increased postoperative complications and poorer survival in surgical oncology, emergency general surgery and orthopaedic surgery populations (4–6). Furthermore, sarcopenia identified prior to intervention is also associated with poorer allograft outcomes following solid organ transplant and greater morbidity and mortality following trauma (7, 8).

In the context of an ageing global population, greater prevalence of comorbidities and increased recognition regarding the role of frailty on postoperative outcomes, there is a greater demand for improved risk stratification and perioperative optimisation of patients to improve surgical outcomes. The identification of sarcopenia as a potential prognostic marker provides an appealing area of intervention. The evidence suggests that early intervention prior to elective surgery can lead to delaying of further loss of muscle mass, as well as offering the potential to treat or even reverse sarcopenia (1). These interventions include a pre-planned package of interventions as part of a multimodal prehabilitation program leading up to the date of surgery, including the delivery of resistance exercise programs lasting at least 4–6 weeks to maintain strength and increase skeletal muscle mass in addition to optimising nutrition (9, 10). Prehabilitation therefore is defined as a clinical strategy where patients undergo both physical and psychological interventions in order to improve their functional capacity, muscle mass and experience of care prior to surgery. Prehabilitation regimens aim to coordinate and deliver these interventions, in addition to other services such as psychological support (11). Recent studies evaluating prehabilitation have shown promising results, with the potential for reducing postoperative complications by up to 50% in addition to other co-benefits in reducing length of stay, reducing disability and enhancing patient wellbeing (12, 13). It is postulated these improved outcomes are due to improved muscle mass and nutritional status of patients that allows improved muscle function and therefore improved functional capacity, surgical resilience, fitness and recovery (14, 15). In evaluating muscle function further, the role of resistance exercise is particularly important in deriving these outcomes, given its benefits in augmenting mitochondrial function and muscle vascularisation that therefore lead to improved muscular function and respiratory capacity (16, 17).

However, not all patients achieve a response after undergoing prehabilitation (18). In part, this may be due to poor access to these programs or the heterogeneity of these programs across jurisdictions as well as the need for emergency surgery that precludes sufficient time for adequate prehabilitation. With further regards to timing of surgery, these prehabilitation regimens are ideally designed to be implemented prior to elective surgery, given the lead-time required to institute a formal training protocol prior to their operation, which can sometimes take weeks. Therefore, it would not be feasible to implement these protocols for patients awaiting emergency surgery due to the time-critical nature of their disease. Additionally, the evidence surrounding prehabilitation remains in its infancy, with further trials required to build robust evidence regarding best-practice approaches (19). Currently, the paradigm behind prehabilitation involves multidisciplinary multimodal interventions including nutrition counselling and support, psychological support and exercise. There are also emerging insights into the role of specific considerations into hormonal balance and chronic inflammation as an extension of this practice. However, there remains a lack of standardisation of prehabilitation protocols, particularly related to the resistance training aspect of these programs. In particular, there are no current best-practice recommendations or standardised frameworks of the modality and method of resistance exercise and how these should be integrated into current prehabilitation methodology. This narrative review therefore seeks to evaluate current insights into resistance training for the purpose of sarcopenia prehabilitation in patients undergoing elective surgery. In doing so, this review aims to integrate these findings within the current standard of multimodal prehabilitation.

## 2 Current perspectives on prehabilitation

Prehabilitation has emerged more recently as a crucial step in the optimisation of surgical outcomes for sarcopenic patients (14). This is largely explained by the greater recognition of sarcopenia as a potential predictor of postoperative complications. Currently, the method of delivering multimodal prehabilitation regimens is variable. One model of care provision is through a prehabilitation service which is often led by an experience prehabilitation or perioperative physician. Due to this, anaesthetists or general medical physicians often assume the role as the coordinator of medical care. In conjunction with this are other vital members including exercise physiologists or physiotherapists for exercise, dietitians and nutritionists for nutritional support, psychologists for mental health support as well as other key stakeholders in patient care such surgeons, speech pathologists, social workers and clinical nurse consultants. These programs are often coordinated within outpatient clinic settings within hospitals where surgeries will take place and where patients are able to access multidisciplinary and specialist care. However, given the need for exercise and nutrition that extends outside of the hospital, often patients will follow-up with community allied health and exercise facilities closer to home for ongoing prehabilitation. How these programs manifest therefore are highly dependent on the resources available, which in turn are affected by social determinants of health including locality and funding.

Furthermore, the ideal type of prehabilitation regimen however remains poorly characterised, with a high degree of heterogeneity in the design of prehabilitation interventions on the background of other confounding variables such as comorbidities, age, functional limitations of the patient and type of surgery (20). However, the overall consensus derived from enhanced recovery after surgery (ERAS) protocols is that in addition to resistance training regimens, evidence-based prehabilitation programs should encompass multimodal interventions that optimise nutrition (21). Additionally, more recent evidence in augmented prehabilitation has also considered the additional role of mental health interventions as well as the management of hormonal balance and chronic inflammation to optimise patient outcomes (22). The literature supports durations of 4–6 weeks to achievea demonstrable effect on muscular physiology and fitness (23). Despite these considerations, there is poor consensus surrounding what the ideal prehabilitation regimen looks like, particularly when considering optimal resistance exercise regimens. Explanations for this include the complexities surrounding implementing and maintaining exercise programs, particularly due to patient factors (e.g., mobility, comorbidities, exercise tolerance), program factors (e.g., heterogeneity based on experience of the trainer) and socioenviromental factors (e.g., access to equipment, access to specialised staff, funding). Therefore, there is a need to characterise the key principles that prehabilitation programs focused on sarcopenic patients should consider when it comes to resistance exercise. In doing so, this will allow clinicians to tailor such models to the specific patient in a way that is safe and effective. Herein, we discuss the key evidence-informed principles of resistance exercise for prehabilitation and briefly provide an up-to-date overview of the role of nutritional counselling to provide a holistic view of the prehabilitation process.

## 3 Resistance exercise

Increased cardiopulmonary reserve is a predictor of improved postoperative outcomes, with many current prehabilitation programs focussing on aerobic exercise to increase the anaerobic threshold of patients preoperatively (24–28). Aerobic exercise targeting 50% of maximal heart rate, calculated as 220—age (in years), three times per week for up to six weeks preoperatively have been shown to successfully improve functional exercise capacity in patients prior to major surgery (25–28). These parameters are a rough guide, with more accurate measures of maximum heart rate being reported including the Tanaka equation [208–0.7×age (in years)] and the Gelish equation [207−0.7 × age (in years)] (29, 30). More accurate measures include using treadmill or bicycle testing, given the variability of maimum heart rate that arises depending on activity (e.g., running or cycling) (31). Resistance training forms a part of these protocols, but the effect of prehabilitation protocols on muscle mass, as well as the types of resistance exercises being delivered is poorly defined. With lower muscle mass being a predictor of poorer postoperative outcomes, interventions aimed at increasing muscle mass should be regularly adopted in prehabilitation protocols for patients with sarcopenia (32, 33). Principles that need to be adhered to when implementing resistance training within a prehabilitation program include training volume, frequency, intensity, and exercise type (34). Furthermore, nuanced consideration needs to be applied to the level of experience of the patient. Specifically, a resistance exercise novice would have differing needs to that of a patient who is well versed in resistance exercise.

### 3.1 Training volume

Training volume is defined as the total amount of work done, and is often expressed as the number of repetitions per exercise across a period of time (either a session, or the entire prehabilitation period) (35). A dose-response relationship between muscle mass and training volume is seen, with more sets per exercise in each session leading to increased muscular hypertrophy (36). There exists a plateau above which increased volumes of training yields minimal gains; training in volumes of 2–3 sets has been shown to be superior compared to a single-set, and is also non-inferior to training in volumes of 4–6 sets (37). When a minimum threshold for mechanical tension is achieved, exercise-induced metabolic stress induces muscle protein accretion (38). This metabolite buildup is seen to be higher in multi-set protocols compared to single-set protocols, further suggesting that multi-set training induces greater levels of hypertrophy (39). Thereby a rationale exists to argue that multi-set protocols up to three sets per exercise should take precedence over single-set protocols when implementing resistance training into prehabilitation programs.

### 3.2 Training frequency

Frequency refers to the number of training sessions undertaken in a given period of time, usually one week, and is closely related to volume when assessing principles of resistance training to induce muscle hypertrophy. It is postulated that untrained individuals can maximise muscle hypertrophy with frequencies of between 2 and 3 times per week per muscle group (40). When equated for volume, higher frequencies of training does not equate to more muscular hypertrophy (41). But when not equated for volume, there is a benefit to increased frequencies of training muscle groups twice weekly, demonstrating statistically increased muscle mass over a fixed period of time (41). However there is scarce data when looking at training frequencies of three or more times per week, although there exists the concept of overtraining if a high frequency of training is performed for a sustained period of time ultimately leading to a rapid decline in performance (42). To avoid this, periodisation of frequency is often undertaken in strength training by alternating between periods of higher and lower frequency training, to avoid potential overtraining (34). In sarcopenic patients undertaking resistance training for muscular hypertrophy in a short period of time of up to six weeks preoperatively, it is unlikely that this threshold for overtraining will be attained. Regardless, frequencies of up to twice weekly per muscle group have low risk of overtraining and remains the best evidence-based threshold for maximising muscular hypertrophy (41).

### 3.3 Training intensity

Training intensity in the form of progressive overload is considered to be the cornerstone in achieving a muscle hypertrophy response (43, 44). Intensity can be varied based on two factors; the percentage of the one-repetition maximum weight of the patient for a given exercise, and the number of repetitions performed. High resistance load with a moderate number of repetitions has traditionally been considered to optimise muscle gains (44). The American College of Sports Medicine guidelines recommend an intensity of 60%–80% of the patient's one-repetition maximum for 8–12 repetitions per set in older adults, especially those with comorbid conditions limiting physical function (44). More recent evidence suggests that more important than weight and number of repetitions is training to failure (45, 46). Participants training with high and low resistance loads are seen to have similar gains in muscular hypertrophy, but with the caveat that momentary muscular failure is the set endpoint for training (45). Use of low resistance loads in training requires higher a higher number of repetitions and therefore time, and is often associated with higher levels of discomfort. Periodisation of intensity can be utilised to modify the intensity and is done in either a linear or undulating model, theorising that training is planned in a manner that avoids overtraining and minimises injury risk (47). A linear periodisation model increases intensity and therefore decreases volume over time, whereas an undulating model varies intensity and volume on a more regular basis. When equated for volume, there is no advantage gained by one method of periodisation over another, confirming the importance of the role in training volume in a program focussed on hypertrophy (36, 48). Therefore, in a time-constrained prehabilitation setting, training programs may implement a form of periodisation to minimise risk of injury whilst keeping training volume high to maximise muscle hypertrophy.

### 3.4 Exercise selection

Exercises utilising eccentric contraction are superior in inducing muscular hypertrophy compared to concentric exercises, theoretically due to increased mechanical stresses placed on muscle bodies, a more rapid protein synthesis response, and stronger anabolic signalling (49, 50). Patients are able to load more weight using eccentric exercise compared to concentric exercise, and therefore there is a greater volume of training performed with the use of eccentric exercise that may be responsible for the increased muscle growth seen with these training protocols (51). These hypertrophic gains are seen to be distributed unevenly in the distal portions of the muscle, as opposed to concentric exercises distributing muscle mass into the middle of muscle bellies (49). The practical implication of this finding is unknown, but overall an eccentric-only model is unlikely to yield a benefit in the prehabilitation setting, especially when considering the greater effect of delayed onset muscle soreness associated with eccentric exercises that would affect overall training volume and intensity (51). Therefore, exercise selection should prioritise yielding the most benefit with respect to hypertrophy that is suited to the patient. Ideally, isotonic exercises where the muscle length changes (such as with squats, bicep curls, tricep extensions) and therefore incorporating both eccentric and concentric contraction are more useful than isometric exercises where the muscle length remains constant (such as with wall sits or plank) (52). When selecting isotonic exercises for hypertrophy, clinicians and practitioners should consider what is practical in terms of mobility, resource access and safety for patients. More experienced patients may be suited to free-weight compound isotonic exercises such as the deadlift or squat however more novice patients, particularly with balance issues, may be more suited to machine-assisted isotonic movements such as leg extensions or bicep curls. Careful resistance exercise programming should include judicious involvement of an experienced physiotherapist, exercise physiologist and/or personal trainer.

Novel approaches such as the implementation of blood flow restriction (BFR) based exercises with lower weights are hypothesised to result in as much muscular hypertrophy as their heavy-weight based exercises (53). These exercises may be better suited to elderly participants in prehabilitation protocols that may not necessarily be able to undertake high intensity and high-weight based exercises with ease, and subsequently would be at higher risk of injury to themselves (54). BFR training is thought to induce increased muscular hypertrophy by inducing a hypoxic environment where muscles produce inflammatory cytokines that stimulate growth factor production. Training is performed with a mechanical torniquet applied to the limb proximal to the muscle that is being trained and inflated to a pressure that produces complete venous occlusion and partial arterial occlusion (55). While there is evidence in healthy populations surrounding the benefits of BFR training, the efficacy of its utility in sarcopenic populations still requires further robust research (54).

### 3.5 Injury prevention

Injury prevention is a key factor in resistance training. The cohort of patients with sarcopenia are often comorbid and frail. Therefore, these patients are at higher risk of injury and subsequent setback in their prehabilitation journey. Furthermore, there may be differing levels of familiarity and experience with resistance training among sarcopenic cohorts. Therefore, multidisciplinary teams involved in prehabilitation programs need to ensure that patient-specific steps are taken to reduce the risk of injury during exercise and to tailor programs towards the skill level of the patient. TParticularly for novices, initial training should occur in a safe and supervised environment, with the patient being near a firm support such as a wall, rail, or even being seated (56). Supervision can be provided by physiotherapists, exercise physiologists or personal trainers in a group setting, or on an individual bases. If training is undertaken at home, an experienced family member may adopt this role to reduce the burden on the healthcare system. Single-joint exercises have been shown to produce a similar hypertrophic effect as multi-joint or compound exercises, and additionally pose a lower risk of injury to the patient since movements are simpler and less joints are loaded simultaneously (57–59). Suboptimal movement patterns should also be monitored and exercises appropriately modified to prevent injury in patients that are unable to adequately perform the exercise (58). Overall, injury prevention strategies are ultimately guided by the patient and their relative restrictions and experience. Clinicians and practitioners therefore have a duty to develop strategies that are specific to the patient after careful consultation.

### 3.6 Barriers to implementation of resistance exercise protocols

Barriers to implementation of resistance training into prehabilitation programs involve both healthcare system factors and patient factors (60, 61). Healthcare resources are limited, and the inclusion of a protocol requiring individualised programming for each patient would require additional personnel to implement effectively. Additionally, physical space such as a gymnasium in which patients can undertake their exercise would need to be made available. Sarcopenic patients are often also sedentary and may have background psychological factors that would impact their motivation to engage with exercise (62). Gym exercisers that perceive instructors to be supportive are more likely to have ongoing engagement with exercise. This is in the form of supporting autonomy (i.e., freedom of exercise choice), competence (i.e., provision of positive feedback and encouraging learning), and relatedness (i.e., provision of emotional support), and has been shown to improve adherence to exercise programs (63). Patient motivation is often also improved by seeing progression in training in the form of results, as well as engagement with like-minded individuals that may be undergoing similar circumstances (60). Introduction of group exercise sessions both reduces the demand for personnel, as higher trainer-to-patient ratios may be utilised, and also encourages engagement between patients and therefore driving motivation.

Ultimately, the ideal training protocol for a patient with sarcopenia will vary, based on patient ability and the resources available. Training volume and intensity should be maximised, whilst utilizing a combination of eccentric and concentric exercises, performed between 3 and 5 times per week (37, 41, 45, 46, 48, 51). Exercise physiologists and physiotherapists involved in prehabilitation should be cognisant of patient ability and ensure exercises are performed in a safe manner to prevent injury (58).

## 4 Nutrition

Nutrition plays a key role in enabling an anabolic environment for muscle hypertrophy following resistance exercise. Well informed nutritional programs as part of a prehabilitation regimen should consider the multidisciplinary involvement of clinicians, dietitians and nutritionists where possible (64). Patients with sarcopenia, as well as those prone to sarcopenia such as the elderly population, experience impaired muscle protein synthesis due to negative protein balance and reduced anabolic responses following meals (65). Therefore, central to nutritional components of prehabilitation regimens is the optimisation of protein intake. This is particularly necessary in the context of resistance exercise aimed at inducing hypertrophy for sarcopenic patients. For such surgical patients, the European Society for Clinical Nutrition and Metabolism (ESPEN) guidelines suggest a minimal intake of 1.2 g of protein per kilogram of bodyweight (66, 67). Satiety remains an issue in meeting these targets and therefore the consideration of nutritional supplements is considered to allow patients to meet these guidelines more effectively (68, 69). Furthermore, randomised-controlled trials have also suggested that patients achieve a higher skeletal muscle index with nutritional supplementation interventions compared to nutritional advice alone (70). More recently, emerging research has identified further nutritional interventions that may ameliorate the deleterious pathophysiological mechanisms that drive sarcopenia. In particular, there is suggestion that the incorporation of specific amino acids in the diet, including the branched chain amino acids (leucine, isoleucine, valine) and cysteine, which influence anabolic signal transduction at multiple points of regulation (71, 72). Leucine supplementation in particular has been shown to counteract inflammatory catabolism processes by upregulating anabolic signalling through pathways including the phosphoinositol 3-kinase signaling pathway (71).

Additionally, optimisation of micronutrients including vitamin D and iron have also been associated with reducing muscle atrophy pathways and improving postoperative outcomes respectively (73–75). Similar findings have also been demonstrated following supplementation with omega-3 fatty acids and vitamin B12 (76, 77). Vitamin D deficiency has also been shown to contribute to sarcopenia development by increasing oxidative stress and attenuate mitochondrial biogenesis and function, and therefore repletion of Vitamin D would modulate these pro-inflammatory responses and reduce muscular atrophy (73). Micronutrient supplementation of trace elements such as selenium and magnesium may also improve postoperative outcomes in patients with sarcopenia who are malnourished (78). Dietary collagen is also thought to stimulate muscle connective tissue synthesis and thereby increase muscle mass given its high glycine, proline and hydroxyproline content (78, 79). However, the ideal requirements of these levels to efficaciously and practically derive benefit in reducing sarcopenia remain unknown. Despite the evidence behind these aforementioned nutritional recommendations, best-practice approaches to nutritional intervention in prehabilitation to address sarcopenia should be tailored towards the patient. In essence, there is a demand for personalised nutritional counselling and intervention that takes into account the needs, dietary requirements, culture, and status of the patient.

Omega-3 fatty acids, or in the form of fish oil, has a role in increasing lean body mass, skeletal muscle mass, which may slow the effects of sarcopenia (80). When older patients were supplemented with fish oil for 6 months, there was associated increases in thigh muscle volume, handgrip strength and 1-RM muscle strength (80). The exact mechanisms underlying these changes are not well understood but likely involve changes in both anabolic and catabolic pathways. It is unclear whether shorter term regimes pre-operatively would have similar effects on those with sarcopenia preoperatively. Another consideration is the theoretical antiplatelet effect of omega-3 fatty acid supplementation, whereby components of PUFA compete with arachidonic acid for incorporation into platelet membranes or cyclooxygenase-mediated pathways (81). Thus, reducing production of arachidonic acid-derived prothrombotic metabolites including thromboxane A2, increase production of antithrombotic metabolites and reduce platelet activation and aggregation. However, there is little clinical evidence that supports a significant effect on perioperative bleeding. In a secondary analysis of the OPERA trial, fish oil did not lead to increased perioperative bleeding (82).

## 5 Future directions

Enhancing the understanding of the management of sarcopenia forms a crucial component in effective prehabilitation of patients identified with this risk factor prior to surgery. The appropriateness and accuracy of sarcopenia grading as well as the consistency of prehabilitation protocols form some of the most important areas for future research.

### 5.1 Diagnosis and grading

One of the most important areas of focus should be on the standardisation of imaging and diagnostic tools for sarcopenia. Current diagnostic methods including DEXA (Dual-Energy x-Ray Absorptiometry) and BIO (BIOelectrical impedance analysis) are widely used, but have limitations regarding accessibility and accuracy (9). Future research should focus on developing more precise and accessible imaging such as through use of CT or MRI imaging to provide more consistent, detailed insights into muscle composition, aiding in the early detection and monitoring of sarcopenia grading (1). Specific biomarkers may also be used in the diagnosis and monitoring of sarcopenia such as the enzymes that play a role in regulating activation of muscle protein synthesis (1, 71). Combined with imaging, these biomarkers can offer a non-invasive and cost-effective means of diagnosing sarcopenia and predicting patient outcomes.

### 5.2 Prehabilitation protocols

The development of evidence-based contemporary prehabilitation protocols should have a focus on standardisation to ensure consistent and effective management of sarcopenia. Current protocols vary widely in terms of exercise types, intensity and duration (9, 64, 83). Future research should aim to identify the most effective components of prehabilitation programs, and standardise these into universally accepted guidelines. Furthermore, factors such as baseline fitness levels, comorbidities and patient preferences should be considered when designing a personalised plan, and the incorporation of technology into these regiments hold significant promise. Wearable devices, mobile health applications, and telemedicine can enhance patient adherence, engagement and monitoring through providing real-time feedback and adjustments, ensuring an optimal level of care tailored to each patients progress and needs (9, 64). Although in its infancy in its application to healthcare, artificial intelligence (AI) could be used in conjunction with wearable smart devices to analyse movement data, identify imbalances and provide feedback to patients (84). Given that AI has the ability to process vast amounts of data including past medical history and assessment results, it could identify at-risk patients for injury and high risk movement patterns, aiding in exercise selection for that individual (85). Generative AI has also been explored to develop personalised exercise programs for mildly medically comorbid pseudo-patients and was able to form safe exercise protocols for these patient scenarios (86). However, recommendations by the application were generic, and essential information such as patients' physical limitations and nutritional state were overlooked. Therefore, prior to practical applications in prehabilitation, generative AI applications need to be further developed to incorporate a more holistic approach to prehabilitation of medically complex patients.

Finally, the full consideration and incorporation of holistic multimodal prehabilitatiom, including the psychological, anti-inflammatory and hormonal interventions is crucial for improving patient outcomes. Future research should focus on integrating psychological support into programs, including interventions such as counselling, stress management workshops and cognitive behavioural therapy and consideration of steroids which ultimately play a significant role in supporting a patient's mood and physiology (20, 64, 83).

## 6 Conclusion

Sarcopenia represents a critical factor in the perioperative care of patients undergoing surgery, significantly impacting postoperative outcomes. Prehabilitation programs that integrate resistance training as a core component can mitigate muscle loss and improve surgical outcomes. Key principles such as maximising training volume, maintaining progressive overload, and utilising a mix of multi-joint and single-joint exercises are foundational in optimizing these programs. Moreover, addressing nutritional deficits, managing chronic inflammation, optimising hormonal balance, and providing psychological support are integral to comprehensive prehabilitation strategies.

Despite the current variability in prehabilitation protocols and the need for further standardisation, the evidence supporting these interventions is robust. Future research should focus on refining diagnostic tools for sarcopenia, standardisation of prehabilitation protocols, and integration of technological innovations to enhance efficacy and patient adherence. By advancing our understanding and implementation of prehabilitation, we can enhance surgical outcomes, reduce postoperative complications, and improve the overall quality of life for patients at risk of sarcopenia.
